# Diabetes Mellitus, Glycemic Traits, and Cerebrovascular Disease: A Mendelian Randomization Study

**DOI:** 10.1212/WNL.0000000000011555

**Published:** 2021-03-30

**Authors:** Marios K. Georgakis, Eric L. Harshfield, Rainer Malik, Nora Franceschini, Claudia Langenberg, Nicholas J. Wareham, Hugh S. Markus, Martin Dichgans

**Affiliations:** From the Institute for Stroke and Dementia Research (M.K.G., R.M., M.D.), Department of Neurology (M.K.G), University Hospital, and Graduate School for Systemic Neurosciences (M.K.G.), Ludwig-Maximilians-University, Munich, Germany; Stroke Research Group, Department of Clinical Neurosciences (E.L.H., H.S.M.), and MRC Epidemiology Unit (C.L., N.J.W.), University of Cambridge, UK; Department of Epidemiology (N.F.), UNC Gillings Global School of Public Health, Chapel Hill, NC; Munich Cluster for Systems Neurology (SyNergy) (M.D.); and German Centre for Neurodegenerative Diseases (DZNE) (M.D.), Munich, Germany.

## Abstract

**Objective:**

We employed Mendelian randomization to explore the effects of genetic predisposition to type 2 diabetes (T2D), hyperglycemia, insulin resistance, and pancreatic β-cell dysfunction on risk of stroke subtypes and related cerebrovascular phenotypes.

**Methods:**

We selected instruments for genetic predisposition to T2D (74,124 cases, 824,006 controls), HbA1c levels (n = 421,923), fasting glucose levels (n = 133,010), insulin resistance (n = 108,557), and β-cell dysfunction (n = 16,378) based on published genome-wide association studies. Applying 2-sample Mendelian randomization, we examined associations with ischemic stroke (60,341 cases, 454,450 controls), intracerebral hemorrhage (1,545 cases, 1,481 controls), and ischemic stroke subtypes (large artery, cardioembolic, small vessel stroke), as well as with related phenotypes (carotid atherosclerosis, imaging markers of cerebral white matter integrity, and brain atrophy).

**Results:**

Genetic predisposition to T2D and higher HbA1c levels were associated with higher risk of any ischemic stroke, large artery stroke, and small vessel stroke. Similar associations were also noted for carotid atherosclerotic plaque, fractional anisotropy, a white matter disease marker, and markers of brain atrophy. We further found associations of genetic predisposition to insulin resistance with large artery and small vessel stroke, whereas predisposition to β-cell dysfunction was associated with small vessel stroke, intracerebral hemorrhage, lower gray matter volume, and total brain volume.

**Conclusions:**

This study supports causal effects of T2D and hyperglycemia on large artery and small vessel stroke. We show associations of genetically predicted insulin resistance and β-cell dysfunction with large artery and small vessel stroke that might have implications for antidiabetic treatments targeting these mechanisms.

**Classification of Evidence:**

This study provides Class II evidence that genetic predisposition to T2D and higher HbA1c levels are associated with a higher risk of large artery and small vessel ischemic stroke.

Cerebrovascular disease is a major public health issue, ranking as the second leading cause of mortality and adult disability worldwide.^1,2^ Type 2 diabetes (T2D) is an established risk factor for cerebrovascular disease.^3,4^ In cohort studies, T2D shows associations with higher risk for both ischemic and hemorrhagic stroke independently of other risk factors.^5^ Also, several studies found associations of measures of hyperglycemia (glycated hemoglobin [HbA1c] and fasting glucose levels) with risk of stroke, both in patients with and without diabetes.^5^ However, large-scale randomized controlled trials (RCTs) testing intensive glucose-lowering in patients with T2D show no significant reductions in risk of stroke, possibly due to insufficient power.^6–8^ Moreover, the effects of T2D or hyperglycemia on etiologic stroke subtypes (large artery stroke, cardioembolic stroke, small vessel stroke, intracerebral hemorrhage) remain elusive.

Currently available antidiabetic medications act by either directly lowering glucose levels or by targeting 2 major mechanisms that contribute to hyperglycemia: insulin resistance or pancreatic β-cell dysfunction.^9^ Observational data suggest that markers of insulin resistance, β-cell dysfunction, and hyperglycemia influence the risk of cardiovascular disease independently of each other.^10,11^ However, data on stroke and its etiologic subtypes are lacking. Moreover, there is a risk of confounding and reverse causation in observational studies. Developing targeted strategies for stroke prevention in patients at risk of or with T2D would require disentangling these relationships.

Mendelian randomization may help to clarify these associations. Mendelian randomization uses genetic variants as instruments for traits of interest and is not prone to confounding and reverse causation.^12^ As such, Mendelian randomization has been proven a powerful methodology for inferring causality.^13,14^ The availability of large-scale genome-wide association studies (GWAS) with detailed phenotyping of cases further enables the exploration of etiologic stroke subtypes that are typically not considered in observational studies.

We leveraged large-scale data from GWAS and performed Mendelian randomization analyses, with the following aims: (1) to examine the effects of genetic predisposition to T2D on risk of ischemic stroke, ischemic stroke subtypes, and intracerebral hemorrhage; (2) to explore the effects of genetically predicted measures of hyperglycemia (HbA1c and fasting glucose levels) on these phenotypes; (3) to examine the associations of genetic predisposition to insulin resistance and β-cell dysfunction with major stroke etiologies; and (4) to explore associations between diabetic traits and related vascular phenotypes including carotid atherosclerosis, neuroimaging markers of white mater integrity, and brain atrophy.

## Methods

### Study Design and Data Sources

This is a 2-sample Mendelian randomization study following STROBE-MR guidelines (Strengthening the Reporting ofMendelian Randomization Studies).^15^ The study is based on publicly available summary statistics from GWAS consortia. Data sources are detailed in table 1. Mendelian randomization uses genetic variants associated with exposures of interest and then explores the associations between the genetic predisposition to this exposure or the genetically predicted levels of the exposure phenotype with disease outcomes. As the genetic predisposition to a trait of interest is not affected by potential confounders, this approach is considered to be less prone to confounding, as compared with traditional observational analyses.

**Table 1:**
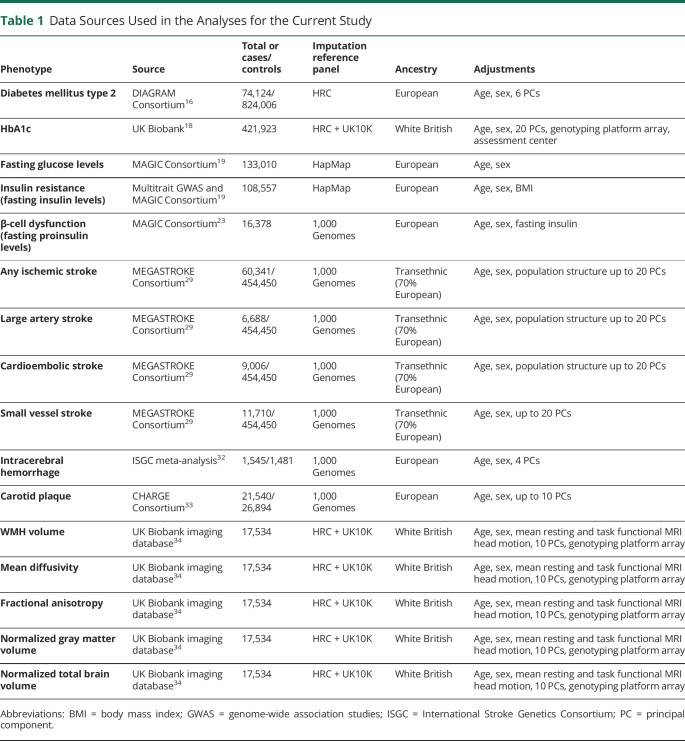
Data Sources Used in the Analyses for the Current Study

Our study design is depicted in figure e-1 and a detailed description of the phenotypes explored as exposures is provided in supplemental table e-1 (doi.org/10.5061/dryad.9s4mw6mdh). We explored associations of genetic predisposition to T2D, measures of hyperglycemia (HbA1c and fasting glucose levels), as well as markers of insulin resistance and β-cell dysfunction with cerebrovascular disease phenotypes including stroke subtypes, carotid atherosclerosis, white matter (WM) integrity, and brain atrophy. Information on genetic variants used as instruments are presented in supplemental tables e-2 to e-7 (doi.org/10.5061/dryad.9s4mw6mdh).

### Genetic Instrument Selection

#### Type 2 Diabetes

We selected genetic instruments from the latest GWAS meta-analysis for T2D based on 74,124 cases and 824,006 controls of European ancestry from 32 studies included in the DIAGRAM consortium.^16^ The analyses were adjusted for age, sex, and population structure. There were 403 distinct genetic variants showing significant associations with T2D in this meta-analysis. We clumped these variants for linkage disequilibrium (LD) based on a distance window of 10,000 kB and an *r*^*2*^ < 0.01 and used the remaining 289 variants as instruments (table e-2, doi.org/10.5061/dryad.9s4mw6mdh). Given the average LD block length of 22,000 kB,^17^ we used a 10,000 kB clumping window, with the notice that we cannot rule out very long-range LD effects.

#### Hyperglycemia

We selected genetic instruments for HbA1c levels (per 1% increment) based on 2 different GWAS that we performed on individuals of White British ancestry in the UK Biobank (UKB).^18^ In the primary analysis, we explored HbA1c levels across the entire range of values among both diabetic and nondiabetic individuals (n = 421,923). In this analysis, we only excluded individuals on antidiabetic medications or insulin at the start of the study (n = 5,468), as these medications affect HbA1c levels beyond genetic influence. In a secondary analysis, we explored HbA1c levels in the prediabetic range among diabetes-free individuals. In this analysis, we excluded individuals with self-reported history of physician-diagnosed diabetes, use of oral antidiabetic drugs or insulin, HbA1c level >6.5%, or random glucose levels >200 mg/dL (n = 400,989). In both analyses, we also excluded 17,534 individuals who were included in the GWAS analysis for imaging phenotypes (see below) to avoid population overlap between exposure and outcome datasets. We adjusted for age, sex, genotyping platform array, assessment center, and the first 20 principal components of the population structure and performed the analyses using BOLT-LMM with correction for relatedness and subtle population stratification. For fasting glucose levels (per 1-SD increment), we used the most recent GWAS meta-analysis (adjusted for age, sex, and population structure) by the MAGIC consortium on 133,010 diabetes-free individuals of European ancestry.^19^ For both HbA1c and fasting glucose, we selected as instruments genetic variants reaching genome-wide significance (*p* < 5 × 10^−8^) after clumping at an *r*^*2*^ < 0.01 threshold (clumping window 10,000 kB). We identified 333 instruments for HbA1c among both diabetic and nondiabetic individuals, 543 instruments for HbA1c levels among diabetes-free individuals, and 21 for fasting glucose levels among diabetes-free individuals (tables e-3 to e-5, doi.org/10.5061/dryad.9s4mw6mdh).

As several variants may influence HbA1c levels through effects on erythrocyte biology and not by inducing hyperglycemia,^20^ to isolate the effects of the hyperglycemia-related genetic component of HbA1c levels, we performed sensitivity analyses excluding those variants reported to be associated at *p* < 0.001 with erythrocyte-related traits (hemoglobin concentration, red blood cell count, hematocrit, mean corpuscular volume, mean corpuscular hemoglobin concentration, mean corpuscular hemoglobin, red cell distribution width, reticulocyte count, reticulocyte fraction of red cells, immature fraction of reticulocytes, high light scatter percentage of red cells, high light scatter reticulocyte count) in Phenoscanner.^21^

#### Insulin Resistance and β-Cell Dysfunction

As instruments for insulin resistance, we used 53 genetic variants identified in a multi-trait GWAS to associate with the 3 components of this phenotype (fasting insulin levels, triglycerides, and high-density lipoprotein cholesterol; table e-6, doi.org/10.5061/dryad.9s4mw6mdh).^22^ All 3 GWAS that were used to perform the multi-trait GWAS were based exclusively on European individuals. We weighted the instruments based on their effects on fasting insulin levels (per 1-log increment) in a GWAS meta-analysis of 108,557 diabetes-free European individuals.^19^ In accordance with existing literature, we proxied β-cell dysfunction based on fasting proinsulin levels (per 1-log increment).^23,24^ We used summary statistics from a GWAS meta-analysis of 16,378 diabetes-free European individuals and identified 21 genetic instruments (at *p* < 5 × 10^−8^, *r*^*2*^ < 0.01; clumping window 10,000 kB; table e-7, doi.org/10.5061/dryad.9s4mw6mdh).^23^ The GWAS for fasting insulin levels was adjusted for age, sex, and population structure,^19^ whereas the GWAS for proinsulin was also adjusted for fasting insulin levels.^23^

We further used T2D-associated genetic variants previously grouped into clusters of diabetic endophenotypes: 3 clusters of insulin resistance (related to obesity, fat distribution, or lipid metabolism) and 2 clusters of β-cell dysfunction, both associated with reduced levels of fasting insulin, but with opposing effects on fasting proinsulin.^25^ We used the clusters of the variants and the respective weights per variant and cluster as described by Udler et al.^25^ (table e-8, doi.org/10.5061/dryad.9s4mw6mdh).

### Proportion of Explained Variance

For all genetic variants used as instruments, we estimated the proportion of explained variance for the respective phenotypes (tables e-2 to e-7, doi.org/10.5061/dryad.9s4mw6mdh). We estimated the variance explained by each genetic variant for T2D based on the method by So et al.^26^ for binary phenotypes and for the continuous traits we used a previously described formula based on summary statistics.^27^ For the estimations regarding T2D, we used a prevalence rate of 8.5%, according to the 2015 estimate of the global prevalence of the disease by the International Diabetes Federation.^28^

### Associations With Outcomes

We then examined associations of the selected instruments with ischemic stroke, ischemic stroke subtypes, and intracerebral hemorrhage (ICH) as the primary outcomes of interest. For ischemic stroke, we used summary GWAS data from MEGASTROKE, mainly consisting of European individuals (70%).^29,30^ We extracted summary GWAS statistics for any ischemic stroke (60,341 cases, 451,210 controls) and for the major ischemic stroke subtypes: large artery stroke (6,688 cases, 238,513 controls), cardioembolic stroke (9,006 cases, 352,852 controls), and small vessel stroke (11,710 cases, 287,067 controls). The major ischemic stroke subtypes in MEGASTROKE were defined according to Trial of Org 10172 in Acute Stroke Treatment (TOAST) criteria.^31^ In sensitivity analyses, we also restricted our analyses to solely individuals of European ancestry. GWAS data for ICH were derived from the International Stroke Genetics Consortium (ISGC) GWAS meta-analysis including 1,545 cases and 1,481 controls of European ancestry.^32^

Presence of carotid plaque, markers of WM tract integrity (WM hyperintensities [WMH] volume, mean diffusivity, fractional anisotropy), and markers of brain atrophy (gray matter volume, total brain volume) were explored as secondary outcomes. Carotid plaque data were derived from a GWAS meta-analysis (21,540 cases, 26,894 controls of European ancestry) from the CHARGE consortium.^33^ As detailed in this meta-analysis, carotid plaques across the individual studies was defined by atherosclerotic thickening of the common carotid artery wall or the proxy measure of luminal stenosis greater than 25%.^33^ For the imaging phenotypes (WMH volume, mean diffusivity, fractional anisotropy, gray matter volume, total brain volume), we undertook GWAS analyses in the UK Biobank neuroimaging dataset including 17,534 individuals of White British ancestry based on the MRI sequences.^34^ In this analysis, we excluded study participants who reported having received a diagnosis of dementia, Alzheimer disease, Parkinson disease, or any other chronic degenerative neurologic problem, demyelinating diseases, brain cancer, nervous system infection, brain abscess, encephalitis, cerebral palsy, head or neurologic injury/trauma, brain hemorrhage, cerebral aneurysm, or stroke (n = 388). We performed linear regression analyses (additive models) for ln-transformed WMH volume, the first principal components of all measurements of mean diffusivity and fractional anisotropy across the different white matter tracts in the diffusion sequences, and for normalized gray matter and total brain volumes. Adjustments were made for age, sex, mean resting and task functional MRI head motion, the genotype platform array, and the first 10 principal components of the population structure.

### Statistical Analysis

All analyses were performed in R (v3.5.0; The R Foundation for Statistical Computing) using the MendelianRandomization, TwoSampleMR, and MR-PRESSO packages.

#### Main Analyses

We applied 2-sample Mendelian randomization using association estimates derived from the abovementioned sources. Following extraction of the *single nucleotide polymorphism* *(SNP)–*specific association estimates between the instruments and the outcomes, and harmonization of the direction of estimates by effect alleles, we computed Mendelian randomization estimates for each instrument with the Wald estimator. We calculated standard errors with the Delta method. We then pooled individual Mendelian randomization estimates using random-effects inverse-variance weighted (IVW) meta-analyses.^35^ For the main analyses, we corrected for multiple comparisons with the false discovery rate approach and set statistical significance at *q* value < 0.05. Associations not reaching this threshold, but showing an unadjusted *p* < 0.05, were considered of nominal significance.

#### Assessment of Pleiotropy and Sensitivity Analyses

Mendelian randomization estimates derived from the IVW approach could be biased in the presence of directional horizontal pleiotropy. As a measure of overall pleiotropy, we assessed heterogeneity across the SNP-specific Mendelian randomization estimates in the IVW Mendelian randomization analyses with the Cochran Q statistic (statistical significance set at *p* < 0.05).^36^ We applied alternative Mendelian randomization methods that are more robust to pleiotropic variants. The weighted median estimator allows the use of invalid instruments as long as at least half of the instruments used in the Mendelian randomization analysis are valid.^37^ The MR-Egger regression allows for the estimation of an intercept term that can be used as an indicator of unbalanced directional pleiotropy.^38^ MR-Egger provides less precise estimates and relies on the assumption that the strengths of potential pleiotropic instruments are independent of their direct associations with the outcome.^38^ The intercept obtained from MR-Egger regression was used as a measure of unbalanced pleiotropy (*p* < 0.05 indicated significance).^38^ Finally, MR-PRESSO regresses the SNP outcome estimates against the SNP exposure estimates to test for outlier SNPs.^39^ Outliers are detected by sequentially removing all variants from the analyses and comparing the residual sum of squares as a global measure of heterogeneity (*p* < 0.05 for detecting outliers); outliers are then removed and outlier-corrected estimates are provided. MR-PRESSO still relies on the assumption that at least half of the variants are valid instruments.^39^ Finally, when significant results were found, we also applied bidirectional Mendelian randomization analyses to test for any inverse associations using diabetes and glucose-related traits as outcomes and stroke subtypes as exposures. For these analyses, due to the low number of SNPs associated with stroke or stroke subtypes, we lowered our *p* value threshold for selecting genetic instruments at *p* < 10^−6^.

### Primary Research Question/Classification of Evidence

Is genetic predisposition to T2D and hyperglycemia associated with the risk of stroke subtypes? This study provides Class II evidence that genetic predisposition to T2D and higher HbA1c levels are associated with a higher risk of large artery ischemic stroke (odds ratio [OR] per 1-log increment in T2D odds: 1.22, 95% confidence interval [CI] 1.17–1.28; OR per 1% increment in HbA1c levels: 2.06, 95% CI 1.60–2.66) and small vessel ischemic stroke (OR per 1-log increment in T2D odds: 1.18, 95% CI 1.13–1.23; OR per 1% increment in HbA1c levels: 1.85, 95% CI 1.50–2.27).

### Standard Protocol Approvals, Registrations, and Patient Consents

This study, conducted in accordance with the STROBE-MR criteria,^15^ was based on publicly available summary statistics from GWAS meta-analyses of individual studies that had already obtained ethical review board approvals and that had obtained written informed consent from all included patients or their guardians.

### Data Availability

This study was based on summary statistics. Data sources are detailed in table 1. The data from the GWAS studies for ischemic stroke, ICH, and glycemic traits are publicly available and may be accessed through the MEGASTROKE,^40^ ISGC,^41^ and MAGIC^42^ web sites, respectively. Data from the UK Biobank GWAS for the neuroimaging traits may be accessed through an application to the UK Biobank. Data for the carotid plaque phenotype may be accessed through an application to the CHARGE Consortium. Detailed information on the genetic variants used as instruments to produce the presented results are available as supplementary material (tables e-2 to e-8, doi.org/10.5061/dryad.9s4mw6mdh).

## Results

The 289 genetic variants used as genetic instruments for T2D explained 12.7% of the variance in T2D prevalence (table e-2, doi.org/10.5061/dryad.9s4mw6mdh), whereas variants used as instruments for the continuous hyperglycemia traits, insulin resistance (proxied by fasting insulin levels), and β-cell dysfunction (proxied by fasting proinsulin) explained lower proportions of variance: 2.6% for HbA1c among both diabetic and nondiabetic individuals, 1.9% for HbA1c among nondiabetic individuals, 1.5% for fasting glucose, 0.7% for insulin resistance, and 4.5% for β-cell dysfunction (tables e-1 to e-5).

### Genetic Predisposition to T2D and Risk of Stroke

**Figure 1:**
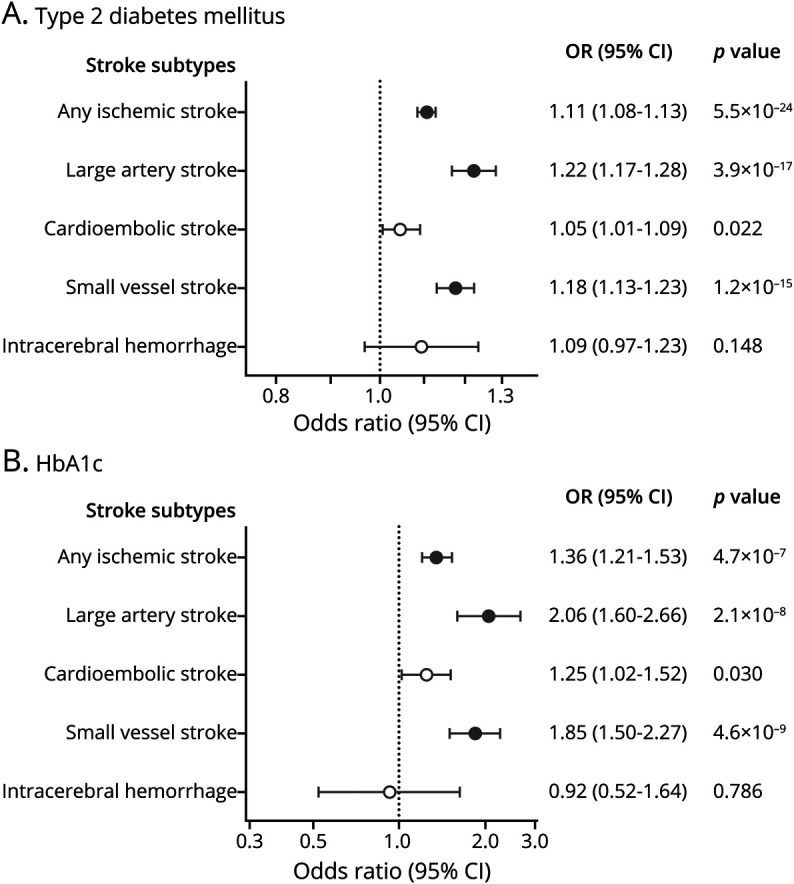
Mendelian Randomization Associations of Genetic Predisposition to Type 2 Diabetes Mellitus and HbA1c Levels Among Diabetic and Nondiabetic Individuals (A) Type 2 diabetes. (B) HbA1c levels. Results derived from random-effects inverse-variance weighted analyses. Full circles correspond to statistically significant association estimates at a false discovery rate–adjusted *p* value < 0.05. CI = confidence interval; OR = odds ratio.

In the primary IVW Mendelian randomization analyses, genetic predisposition to T2D (1-log increment = 2.72-fold higher odds) was significantly associated with a higher risk of any ischemic stroke (OR 1.11, 95% CI 1.08–1.13), large artery stroke (OR 1.22, 95% CI 1.17–1.28), and small vessel stroke (OR 1.18, 95% CI 1.13–1.23; figure 1A). In addition, there was an association of nominal significance with higher risk of cardioembolic stroke (OR 1.05, 95% CI 1.01–1.09), but no significant association with ICH (OR 1.09, 95% CI 0.97–1.23; figure 1A). With the exception of ICH, there was evidence of significant heterogeneity in all of the main analyses (*p* < 0.05; table e-9, doi.org/10.5061/dryad.9s4mw6mdh), but no evidence of unbalanced pleiotropy, as assessed by the Egger intercept *p* values (all *p* > 0.05; table e-10). Across sensitivity analyses based on alternative Mendelian randomization methods (weighted median, MR-Egger, outlier-corrected MR-PRESSO), all effects remained directionally consistent and all estimates stable with *p* < 0.05 for any ischemic stroke, large artery stroke, and small vessel stroke (table e-10). Similar results were also obtained when restricting the analyses to the European population of MEGASTROKE (table e-10). Bidirectional Mendelian randomization analyses showed no effect of genetic predisposition to any ischemic stroke, large artery stroke, or small vessel stroke on risk of T2D (table e-11).

### Genetic Predisposition to Measures of Hyperglycemia and Risk of Stroke

In analyses of hyperglycemia traits, we found that genetically predicted HbA1c levels (per 1% increment) were significantly associated with risk of any ischemic stroke (OR 1.36, 95% CI 1.21–1.53), large artery stroke (OR 2.06, 95% CI 1.60–2.66), and small vessel stroke (OR 1.85, 95% CI 1.50–2.27; figure 1B). There was evidence of heterogeneity in the analyses for HbA1c levels (table e-8, doi.org/10.5061/dryad.9s4mw6mdh) and in some alternative Mendelian randomization analyses the effect estimates for any ischemic stroke, large artery stroke, and small vessel stroke were smaller (table e-8). However, in sensitivity analyses that excluded SNPs influencing HbA1c levels through erythrocyte-related traits, the association estimates were even larger (ischemic stroke: OR 1.53, 95% CI 1.35–1.75; large artery stroke: OR 2.83, 95% CI 2.06–3.89; small vessel stroke: OR 2.26, 95% CI 1.72–2.97; table e-10, doi.org/10.5061/dryad.9s4mw6mdh) and there was no evidence of heterogeneity (all *p* > 0.10). Similar results were obtained when restricting analyses for stroke subtypes to the European population of MEGASTROKE, as well as when performing analyses for HbA1c in the nondiabetic range among diabetes-free individuals (figure e-2; table e-10). In bidirectional Mendelian randomization analyses, genetic predisposition to any ischemic stroke, large artery stroke, or small vessel stroke was not associated with HbA1c levels (table e-11). In contrast, we found no significant associations between genetically predicted fasting glucose levels among diabetes-free individuals and risk of stroke subtypes (figure e-2; table e-10).

### Genetic Predisposition to Insulin Resistance, β-Cell Dysfunction, and Risk of Stroke

**Figure 2:**
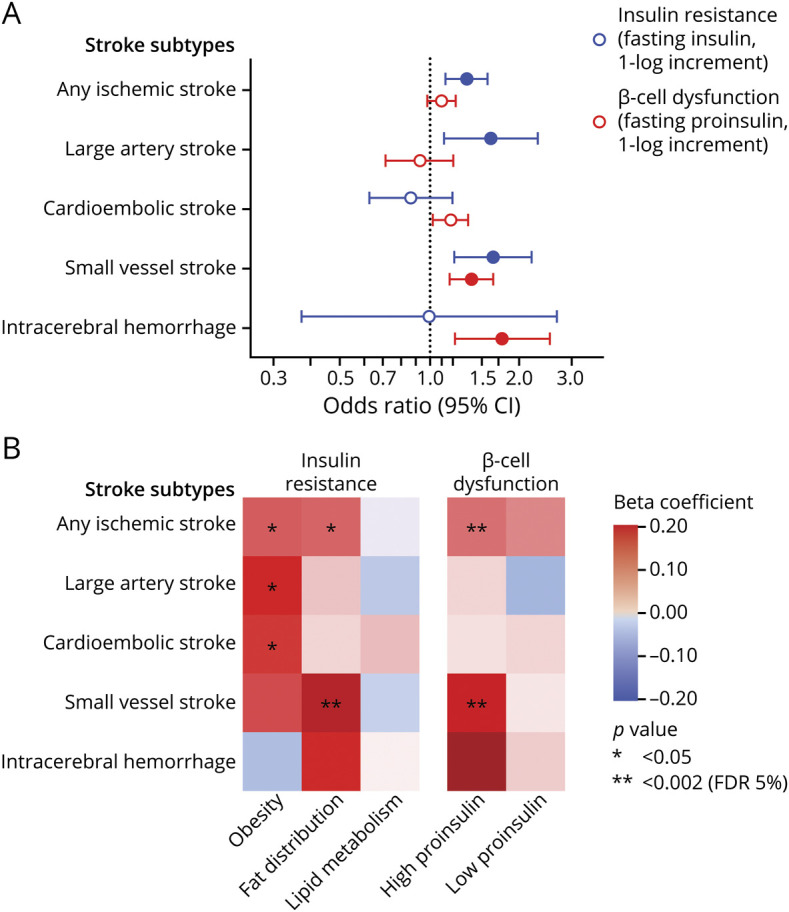
Mendelian Randomization Associations of Genetically Predicted Insulin Resistance and β-Cell Dysfunction With Stroke Subtypes (A) Results derived from random-effects inverse-variance weighted analyses. (B) Heatmap of the associations between clusters of diabetic endophenotypes related to β-cell dysfunction and insulin resistance with the risk of stroke subtypes. Full colored circles in A correspond to statistically significant association estimates at a false discovery rate (FDR)–adjusted *p* value < 0.05. CI = confidence interval.

We next selected genetic variants as instruments for insulin resistance and β-cell dysfunction, the 2 primary underlying mechanisms contributing to the development of hyperglycemia and T2D. Among diabetes-free individuals, we found genetic predisposition to insulin resistance (1-log increment in fasting insulin levels) to be associated with a higher risk for ischemic stroke (OR 1.33, 95% CI 1.13–1.57), large artery stroke (OR 1.60, 95% CI 1.12–2.31), and small vessel stroke (OR 1.63, 95% CI 1.21–2.20; figure 2A). Genetic predisposition to β-cell dysfunction (1-log increment in fasting proinsulin levels) was further associated with a higher risk for small vessel stroke (OR 1.38, 95% CI 1.17–1.63) and ICH (OR 1.75, 95% CI 1.21–2.52). Furthermore, there was an association of nominal significance between genetic predisposition to β-cell dysfunction and the risk of cardioembolic stroke (OR 1.18, 95% CI 1.03–1.35). There was no heterogeneity in these analyses (table e-9, doi.org/10.5061/dryad.9s4mw6mdh) and the results were consistent in alternative Mendelian randomization analyses, as well as in analyses restricted to individuals of European ancestry (table e-10).

To gain additional insights into the relationship among insulin resistance, β-cell dysfunction, and etiologic stroke subtypes, we further explored the effects of T2D-associated variants clustered in 5 different mechanisms of action. These included 3 clusters for insulin resistance (mediated by obesity, fat distribution, lipid metabolism) and 2 clusters related to β-cell dysfunction (associated with high or low proinsulin). In multivariable analyses including all clusters and also adjusting for their effects on HbA1c, we found significant effects of genetic predisposition to β-cell dysfunction related to high proinsulin on risk of ischemic stroke and small vessel stroke (figure 2B). We further found genetic predisposition to insulin resistance mediated through altered fat distribution to be associated with higher risk of small vessel stroke. Genetic predisposition to insulin resistance mediated through obesity showed associations of nominal significance with large artery and cardioembolic stroke.

### Genetic Predisposition to T2D and Glycemic Traits and Associations With Etiologically Related Cerebrovascular Phenotypes

Table 2 presents the Mendelian randomization associations of genetic predisposition to T2D, measures of hyperglycemia, insulin resistance, and β-cell dysfunction with carotid plaque, as well as with neuroimaging traits related to white matter integrity and brain atrophy. Genetic predisposition to T2D and genetically elevated HbA1c levels were associated with carotid plaque. We further found a significant association between genetic predisposition to T2D and lower fractional anisotropy, a diffusion imaging marker of impaired white matter tract integrity, as well as significant associations with lower gray matter and total brain volumes (table 2). Genetic predisposition to β-cell dysfunction (1-log increment in fasting proinsulin levels) was further associated with lower gray matter volume (β −0.13, 95% CI −0.20 to −0.07) and total brain volume (β −0.17, 95% CI −0.23 to −0.11; table 2). These results remained stable in sensitivity analyses (table e-10, doi.org/10.5061/dryad.9s4mw6mdh).

**Table 2:**
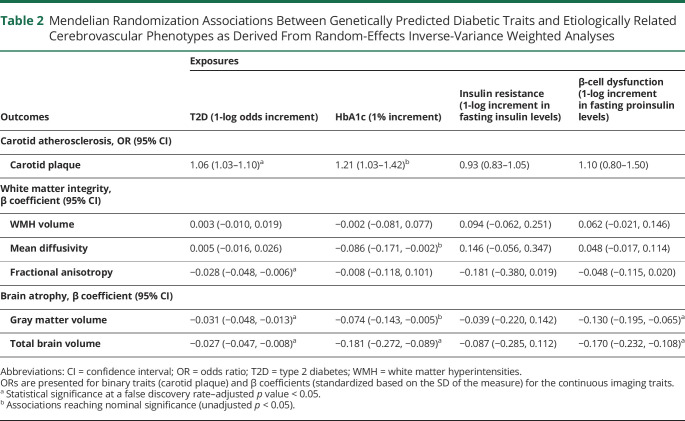
Mendelian Randomization Associations Between Genetically Predicted Diabetic Traits and Etiologically Related Cerebrovascular Phenotypes as Derived From Random-Effects Inverse-Variance Weighted Analyses

## Discussion

Leveraging large-scale GWAS data in Mendelian randomization analyses, we investigated the causal associations between T2D, glycemic traits, and cerebrovascular disease. We found genetic predisposition to T2D and hyperglycemia (elevated HbA1c levels) to be associated with a higher risk of ischemic stroke, particularly large artery and small vessel stroke. Independently of hyperglycemia, genetic predisposition to insulin resistance but not β-cell dysfunction was associated with higher risk of large artery stroke, whereas genetic predisposition to both insulin resistance and β-cell dysfunction was associated with small vessel stroke. Genetic determinats for T2D and hyperglycemia further showed significant effects on carotid plaque and fractional anisotropy, a WM neuroimaging marker related to cerebral small vessel disease, as well as neuroimaging markers of brain atrophy. Furthermore, genetic predisposition to β-cell dysfunction was associated with intracerebral hemorrhage and neuroimaging markers of brain atrophy.

Our Mendelian randomization results provide genetic evidence for a causal effect of T2D and hyperglycemia on risk of ischemic stroke. T2D is among the established risk factors for stroke and vascular disease in general,^4^ but primary prevention trials focusing on intensive glucose control or specific oral antidiabetic agents showed inconsistent effects on stroke risk.^6,8^ Previous Mendelian randomization studies were underpowered to detect effects of hyperglycemia (HbA1c or fasting glucose levels) on stroke risk.^43,44^ Here, by using data from >400,000 individuals from the UKB, we were able to show that genetically elevated HbA1c levels are associated with a higher risk of ischemic stroke, thus suggesting that preventive strategies focusing on long-term HbA1c-lowering will result in risk reductions for ischemic stroke. The lack of significant effects in previous trials might relate to insufficient power due to the low number of incident stroke events, short follow-up periods, and differences in the efficacy profiles of the individual treatments.^45^

We found the effects of genetic predisposition to T2D and hyperglycemia to be specific for large artery and small vessel stroke. In accordance with these results, we found genetic predisposition to T2D to be associated with carotid plaque, an atherosclerotic phenotype, and fractional anisotropy, a marker of WM integrity associated with small vessel disease. Thus, our findings provide evidence for a causal involvement of T2D and hyperglycemia in both large artery atherosclerosis and cerebral small vessel disease. The discordant effects between genetically predicted HbA1c and fasting glucose levels might relate to the fact that HbA1c levels are a more accurate marker of average glucose levels and less prone to between-measurement variability than single measurements of fasting glucose. Differences in sample sizes between the GWAS, as well as the inclusion of nondiabetic patients in the analysis for HbA1c levels, might also partly explain this discordance. On the contrary, we found no significant effects of T2D or other diabetic traits on cardioembolic stroke. Differences in the magnitude of the effects between stroke subtypes might in part explain the heterogeneity in the effects of glucose-lowering treatments across previous clinical trials.^45^ On the basis of our findings, future trials testing glucose-lowering approaches should account for stroke subtypes.

As another finding, we show that genetic predisposition to insulin resistance and β-cell dysfunction influences the risk of stroke. This could have clinical implications for oral antidiabetic medications. Whereas all antidiabetic agents lower glucose levels, some drug classes primarily target insulin sensitivity and others primarily target β-cell function.^9^ Specifically, metformin and thiazolidinediones primarily act by improving insulin sensitivity, whereas drug classes like α-glucosidase inhibitors, sulfonylureas, and GLP1 receptor agonists primarily act by increasing insulin secretion from the β cells.^9^ How these drug classes influence risk of the different stroke subtypes should be explored further in future research.

Our study has several methodologic strengths. The large sample size (898,130 individuals for diabetic traits and up to 514,791 individuals for stroke) and nature of our datasets provided the power to detect differential effects of diabetes on etiologic stroke subtypes and to perform multiple sensitivity analyses for testing the validity of the Mendelian randomization assumptions, thus minimizing the possibility of biased results. Whereas the genetic determinants of HbA1c might influence its levels via both erythrocyte and glycemic biology, we provided support for the latter, as the effects were stronger when focusing on variants not associated with erythrocyte traits. Incorporating insulin resistance and β-cell dysfunction on top of hyperglycemia in the analyses offered deeper insights into the pathophysiologic mechanisms linking diabetes with the different stroke subtypes. Finally, the exploration of additional cerebrovascular disease traits enabled us to triangulate our findings for stroke subtypes by showing similar associations for etiologically related phenotypes.

Our study also has limitations. First, by design Mendelian randomization examines the effects of lifetime exposure to the traits of interest, which might differ from the effects of clinical interventions (e.g., glucose-lowering approaches) applied for shorter time periods later in life. Second, T2D was analyzed as a binary trait and this might violate the monotonicity assumption of Mendelian randomization because only a fraction of individuals with increased genetic liability to T2D will actually get the disease. Thus, genetic liability to T2D that is used as an exposure in our analyses might capture a combination of underlying mechanisms including hyperglycemia, insulin resistance, and β-cell dysfunction. Third, the Mendelian randomization analyses for insulin resistance were weighted based on the effects of the genetic variants on fasting insulin adjusting for body mass index and the analyses for β-cell dysfunction based on the effects of the variants on fasting proinsulin adjusting for fasting insulin. These adjustments in the original GWAS might increase the risk for collider bias in Mendelian randomization analyses,^46^ which should be considered when interpreting our findings. Fourth, the analyses for HbA1c and fasting glucose that were restricted to nondiabetic individuals might also introduce collider bias in the analyses, which might bias the association estimates to the null. Yet the results for HbA1c in the entire population of both diabetic and nondiabetic individuals showed similar results. Fifth, the variance explained by the genetic instruments used for hyperglycemic traits, insulin resistance, and β-cell dysfunction was very low, which might have limited the power of our analyses. However, despite the low proportion of variance explained, the instruments were sufficiently strong, thus ruling out potential weak instrument bias. Sixth, there was high heterogeneity in the majority of the Mendelian randomization analyses performed for this study. Whereas the results from alternative Mendelian randomization methods were consistent, we cannot entirely rule out the possibility of bias in the derived effect estimates due to pleiotropic effects of the genetic instruments. Seventh, ischemic stroke subtypes were defined according to the TOAST classification system, which although widely used, might still inherently lead to misclassifications, especially in cases of mixed stroke etiology. Eighth, many of our exposure phenotypes like HbA1c levels, fasting glucose, and fasting insulin are time-dependent and might change with age, disease stage, and behavioral factors, as well as by epigenetic factors. However, our Mendelian randomization analyses are inherently limited in not taking such effects into account. Novel methods in addressing the time-varying effects^47^ of these phenotypes on stroke subtypes should be examined in the future using datasets with available data. Finally, our analyses were primarily based on datasets involving individuals of European ancestry and might thus not be applicable to other ethnicities.

Our results suggest causal associations of T2D and hyperglycemia with a higher risk for ischemic stroke, particularly large artery and small vessel stroke. Against findings from secondary analyses of clinical trials, our results support that therapeutic approaches aimed at lowering HbA1c have the potential to decrease the risk of ischemic stroke.

## Publication History

Previously published on medRχiv (doi: https://doi.org/10.1101/2019.12.27.19015834). Received by *Neurology* April 8, 2020. Accepted in final form December 23, 2020.

## Glossary

CI: confidence interval
GWAS: genome-wide association studies
ICH: intracerebral hemorrhage
ISGC: International Stroke Genetics Consortium
IVW: inverse-variance weighted
LD: linkage disequilibrium
OR: odds ratio
RCT: randomized controlled trial
SNP: single nucleotide polymorphism
STROBE-MR: Strengthening the Reporting of Mendelian Randomization Studies
T2D: type 2 diabetes
TOAST: Trial of Org 10172 in Acute Stroke Treatment
UKB: UK Biobank
WM: white matter
WMH: white matter hyperintensities
